# Rainfall-driven sex-ratio genes in African buffalo suggested by correlations between Y-chromosomal haplotype frequencies and foetal sex ratio

**DOI:** 10.1186/1471-2148-10-106

**Published:** 2010-04-23

**Authors:** Pim van Hooft, Herbert HT Prins, Wayne M Getz, Anna E Jolles, Sipke E van Wieren, Barend J Greyling, Paul D van Helden, Armanda DS Bastos

**Affiliations:** 1 Resource Ecology Group, Wageningen University, Droevendaalsesteeg 3a, 6708 PB Wageningen, The Netherlands; 2 Mammal Research Institute, Department of Zoology & Entomology, University of Pretoria, Pretoria 0002, South Africa; 3 Department of Environmental Science Policy & Management, University of California at Berkeley, 140 Mulford Hall, CA 94720-3112, USA; 4 Department of Biomedical Sciences, College of Veterinary Medicine, Oregon State University, Corvallis OR 97331, USA; 5 Agricultural Research Council, Irene 0062, South Africa; 6 DST/NRF Centre of Excellence for Biomedical TB Research, US/MRC Centre for Molecular and Cellular Biology, Division of Molecular Biology and Human Genetics, Faculty of Health Sciences, Stellenbosch University, Tygerberg 7505, South Africa

## Abstract

**Background:**

The Y-chromosomal diversity in the African buffalo (*Syncerus caffer*) population of Kruger National Park (KNP) is characterized by rainfall-driven haplotype frequency shifts between year cohorts. Stable Y-chromosomal polymorphism is difficult to reconcile with haplotype frequency variations without assuming frequency-dependent selection or specific interactions in the population dynamics of X- and Y-chromosomal genes, since otherwise the fittest haplotype would inevitably sweep to fixation. Stable Y-chromosomal polymorphism due one of these factors only seems possible when there are Y-chromosomal distorters of an equal sex ratio, which act by negatively affecting X-gametes, or Y-chromosomal suppressors of a female-biased sex ratio. These sex-ratio (SR) genes modify (suppress) gamete transmission in their own favour at a fitness cost, allowing for stable polymorphism.

**Results:**

Here we show temporal correlations between Y-chromosomal haplotype frequencies and foetal sex ratios in the KNP buffalo population, suggesting SR genes. Frequencies varied by a factor of five; too high to be alternatively explained by Y-chromosomal effects on pregnancy loss. Sex ratios were male-biased during wet and female-biased during dry periods (male proportion: 0.47-0.53), seasonally and annually. Both wet and dry periods were associated with a specific haplotype indicating a SR distorter and SR suppressor, respectively.

**Conclusions:**

The distinctive properties suggested for explaining Y-chromosomal polymorphism in African buffalo may not be restricted to this species alone. SR genes may play a broader and largely overlooked role in mammalian sex-ratio variation.

## Background

Sex-ratio (SR) distorters are genes that cause an unequal representation of the sex chromosomes among the offspring through differences in number, quality or function of X- and Y-bearing spermatozoa. These SR distorters are typically located on one of the sex chromosomes. They act during spermatogenesis by distorting the meiosis in their own favour (meiotic drive) or by impairing the function or increasing the mortality of the opposite-sex gametes, which gives the SR distorters a selective advantage [1-3]. Because of this selective advantage, newly mutated SR distorters can quickly invade a population. In principal, SR distorters may exert their effect not only during spermatogenesis but also post-copulation in the female genital tract, by influencing sperm transport, sperm survival or fertilization capability. SR distorters are assumed to be selfish and non-adaptive for their carriers [1,4,5].

SR distorters exert strong selection pressure for suppressors of the distortion, which are genes occurring on the opposite sex chromosome or on one of the autosomal chromosomes, that neutralize the action of the distorter and often lead to a genetic arms race between the two types of SR genes [6]. A stable gene polymorphism is possible when SR-distorter carriers have a lower fitness, thereby preventing the driving distorter alleles from going to fixation [6]. (A SR suppressor does not prevent SR-distorter fixation since the SR distorter still has a selective advantage compared to the wild-type allele, given that it is only being neutralized when co-occurring with a SR suppressor in the same individual.) Most examples of SR distorters are found in *Drosophila *spp. [7]. Their discovery required sophisticated crossing schemes, often between individuals from different populations or incipient species [7]. Although sex chromosomes with SR distorting elements are known in *Drosophila *spp. since 1925, the identity of causal genes remained unknown until the late 1990s because the SR distorting elements are usually associated with complex chromosome inversions, impeding their genetic mapping [7-12]. Nevertheless, SR distorters are thought to be relatively common, even in mammals, but are rarely observed, probably because of rapid gene fixation and SR suppressors that mask their activities [4,5]. (When a SR distorter and SR suppressor are co-occurring in a population both will go to fixation, unless they have a lowered fitness.)

Stable presence of multiple alleles (per locus) or haplotypes (combinations of alleles at different loci along a chromosome inherited en block) on the haploid Y-chromosome in the absence of population subdivision or metapopulation structure only seems possible with negative frequency-dependent selection, since otherwise the fittest allele or haplotype would inevitably sweep to fixation [1,13-15]. SR distorters generally reduce the fitness of their bearer by a reduction of the sperm count making negative frequency-dependent selection possible through associations between mating rate and polygamy on one hand and sperm depletion and sperm competition on the other [1,5,7,16,17]. In this scenario increased SR distorter frequencies result in an increasingly biased population sex ratio and in a decrease of the average semen quality, which can cause an increase in the female or male mating rate. An increased mating rate benefits wild-type alleles at the expense of SR distorters as their carriers perform better in sperm competition (with increased female mating rate) and show less decrease in fertility after repeated matings due to sperm depletion (with increased male mating rate). Also SR suppressors are hypothesised to have a fitness cost considering that they are polymorphic in *Drosophila *spp. [18]. A fitness cost is necessary to prevent gene fixation in view of their selective advantage against wild-type alleles. (Wild-type alleles have a low transmission rate when co-occurring with a SR distorter.) Alternatively, stable gene polymorphism at the Y chromosome may result from interactions in the population dynamics of SR distorters and SR suppressors as indicated in simulation studies, albeit in a small fraction of parameter space [13,19-21].

The African buffalo (*Syncerus caffer*) population of the Kruger National Park (KNP, South Africa, 22°-25° S, 31°-32° E, 20,000 km^2^) consists of some 30,000 individuals [22,23]. The population was completely fenced from neighbouring populations in Zimbabwe and Mozambique between 1976 and 2004. The KNP buffalo are polygamous and live in both small bachelor herds and mixed-sex herds of a few dozen to over 1,000 individuals [24,25]. Reproduction is strongly related to the availability and seasonality of resources with around 75% of all births occurring late in the wet season, from January to March [26]. The foetal sex ratio for the population as a whole does not differ significantly from equality across decades (1969-1998: *P*_exact _= 0.52, sex ratio (male proportion) = 0.506, 95% CI = 0.487-0.526, *n *= 2626) in agreement with "Fisher's Principle", which predicts the evolution of an equal birth sex ratio, assuming equal cost of daughters and sons [27,28].

High Y-chromosomal microsatellite polymorphism, characterized by rainfall-correlated haplotype-frequency shifts between year cohorts, has been observed not only in the KNP population but also in the Hluhluwe-iMfolozi Park (HiP) population located some 300 km farther south (gene diversity *H*, probability of randomly sampling two different alleles or haplotypes; *H *= 0.48 and 0.74 in HiP and KNP respectively; haplotype number = 5 and 15 in HiP and KNP, respectively) [29]. The gene diversity in the KNP population is similar to that recently observed in Eurasian cattle (all breeds combined: *H *= 0.82; mean of the individual breeds: *H *= 0.63), although the estimate for cattle is positively biased due to a larger number of microsatellites (five vs. three in African buffalo) [30]. When looking at the individual microsatellites, gene diversity and allelic diversity are higher in the single African buffalo population of KNP than in all Eurasian cattle breeds combined (African buffalo: *H *= 0.60, 0.66, and 0.67 with 5, 7 and 9 alleles for microsatellites UM1113, UM0304 and INRA189, respectively; cattle: *H *= 0.05, 0.04, 0.36, 0.47 and 0.52 with 2, 3, 4, 4, and 8 alleles for microsatellites INRA124, BM861, BYM1, DYZ1 and INRA189, respectively; supplementary info in [30]).

The observation of high genetic diversity despite yearly haplotype frequency fluctuations was surprising as the latter is expected to quickly result in gene fixation [29]. The presence of multiple haplotypes cannot be attributed to population subdivision. There is no significant genetic differentiation between Gonarezhou NP (Zimbabwe), northern KNP and southern KNP indicating extensive (historical) gene flow (autosomal microsatellites: 95% CI F_ST _= -0.017 - 0.031) [31]. This is supported by radio-tracking data of over 130 individual KNP buffalo that show all sex and age groups move between mixed-sex herds, with monthly emigration rates of 9-26% for adult males and 2% for females and subadults [32]. The high haplotype diversity also cannot be explained by recent mutations as three of the five major haplotypes in KNP (112, 557 and 436; total frequency = 74%) differ by at least seven mutational events (Figure 1 in [29]). The presence of multiple haplotypes therefore indicates some kind of balancing selection. Balancing selection is expected to result in an increased gene diversity relative to haplotype (allele) diversity because of a relatively even haplotype (allele) frequency distribution. This is supported by the observation that Y-chromosomal gene diversity is higher in African buffalo than in Eurasian cattle when comparing microsatellites with a similar number of alleles (*H *= 0.66-0.67 with 5-7 alleles in the KNP buffalo vs. *H *= 0.52 with 8 alleles in cattle; *H *= 0.46 with 2 and 3 alleles for microsatellites UM1113 and UM0304, respectively, in the HiP buffalo vs. *H *= 0.04-0.05 with 2 alleles in cattle).

**Figure 1:**
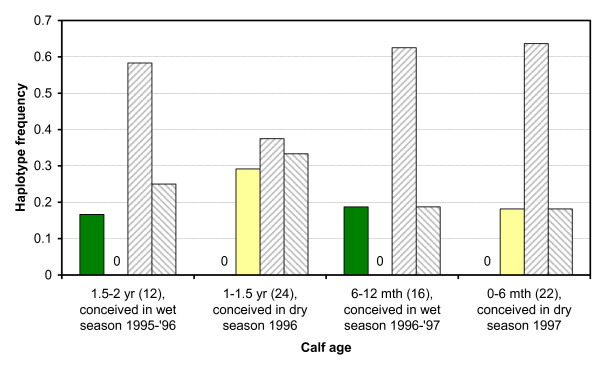
**Seasonal variation in Y-chromosomal haplotype frequencies among calves in relation to period of conception**. Green bars: haplotype 112, Yellow bars: haplotype 557, Upward hatched bars: haplotype 436, Downward hatched bars: other haplotypes. The '0' denotes the absence of haplotype 112 or 557 as applicable. Sample size is given in brackets.

We hypothesise that the frequency shifts in Y-chromosomal haplotypes between year cohorts in the African buffalo population of KNP are indicative of SR genes. Accordingly, we expected to find temporal correlations between Y-chromosomal haplotype frequencies and sex ratios of foetuses and calves (even though the foetal sex ratio for the population as a whole does not differ significantly from equality).

## Results

A temporal genetic analysis of genetic differentiation in calves using Y-chromosomal microsatellite data demonstrated highly significant (*P*_randomisation-based _= 0.00089, *n *= 74) differences between male calves conceived during a dry season (age cohorts from 1998: 0-6 months, 1-1.5 years) and those conceived during a wet season (age cohorts from 1998: 6-12 months, 1.5-2 years). This was mainly due to an opposite (negative) correlation between haplotypes 112 and 557 (Figure 1). Haplotype 557 was observed only among males conceived during the observed dry seasons, whilst haplotype 112 was observed only among males conceived during the observed wet seasons. A negative (112 and 557 in different cohorts) or positive (112 and 557 in the same cohort) correlation of this magnitude by chance for these two haplotypes, either between seasons (wet vs. dry) or years (0-year vs. 1-year old calves), is highly unlikely (*P*_exact _= 0.00022, sum of all four possibilities, *n *= 74).

**Figure 2:**
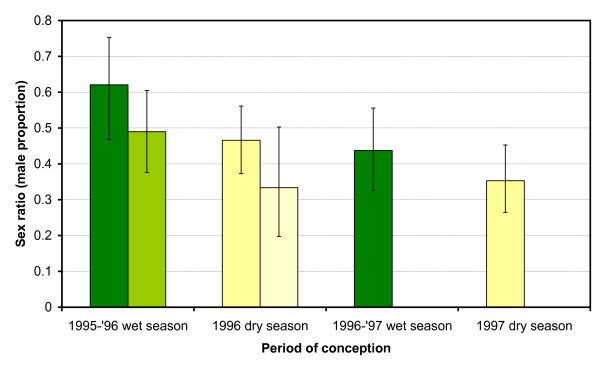
**Seasonal variation in sex ratio among foetuses and calves in relation to period of conception**. Green bars: wet season, Yellow bars: dry season, Dark bars: calves, Light bars: foetuses, Error bars: 90% CI. The age cohorts are the same as those used with the genetic analyses (Figure 1).

**Figure 3:**
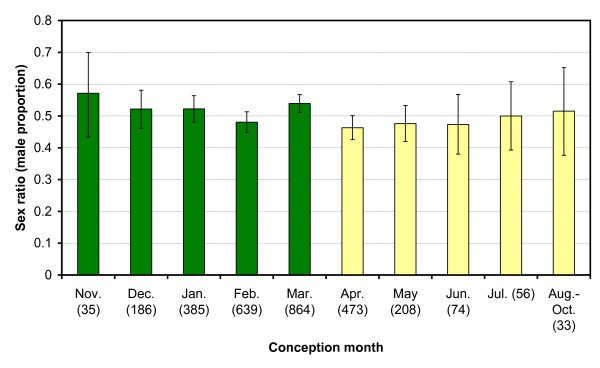
**Mean monthly foetal sex ratios**. Green bars: wet season, Yellow bars: dry season, Error bars: 90% CI. Sample size is given in brackets. Monthly data are pooled across years (1978-1998).

If haplotypes 112 and 557 are linked to SR distorters or SR suppressors then one would expect an alternating seasonal pattern with sex ratios (male proportion) as well, which was indeed observed (Figure 2). In the same age cohorts as were used in the genetic analyses the sex ratio was higher among individuals conceived during the wet seasons than among those conceived during the dry seasons (*P*_exact _= 0.096, *n *= 291; calves culled in 1998 plus foetuses collected in 1996). There was an identical pattern in earlier age cohorts. Among 0-4 months old calves culled between June 1982 and February 1983, the sex ratio was only 0.27 (8/30) among those conceived in the dry season of 1981 (culled in June-November) but as high as 0.69 (9/13) among those conceived in the subsequent wet season (culled in January-February) (*P*_exact _= 0.016) [33]. Most importantly, a significant seasonal difference in sex ratio, with an effect size of 0.045 (absolute difference between two sex ratios), was observed among the foetuses collected between 1978 and 1998 (Figure 3 and Table 1; *P*_exact _= 0.025; data pooled across years). The sex ratio was female-biased during the dry season (*P*_Wilsonscore _= 0.049, one-sided) and male-biased during the wet season (*P*_Wilsonscore _= 0.056, one-sided). The largest difference was observed at the change of seasons between March and April. The seasonal difference in sex ratio was already present among foetuses ≤ 4 months old (Table 1; *P*_exact _= 0.035; data pooled across years), ruling out late-term foetal loss as an explanation.

**Table 1:** Association between foetal age and seasonal foetal sex ratio (male proportion)

Foetal age (mth)	Dry seasons			Wet seasons			***P***_**exact**_**-value** dry vs. wet
	**Mean**	**95% CI**	***n***	**Mean**	**95% CI**	***n***	

2-12	0.472	0.438-0.505	844	0.517	0.496-0.539	2109	0.025
2-4	0.439	0.389-0.492	335	0.514	0.470-0.557	504	0.035
5-12	0.493	0.450-0.536	509	0.518	0.494-0.543	1605	0.33

**Figure 4:**
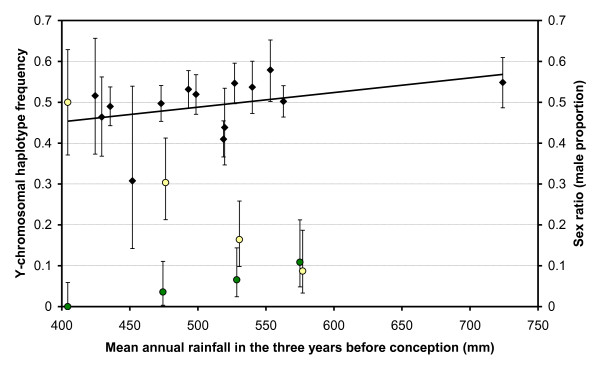
**Correlations of annual foetal sex ratio and Y-chromosomal haplotype frequencies per year cohort with rainfall**. Black diamonds: sex ratio, Green circles: haplotype 112, Yellow circles: haplotype 557, Error bars: 90% CI. Y-chromosomal haplotype frequencies: weighted average across several years for both haplotype frequencies and annual rainfall. Annual foetal sex ratios: years running from Nov. till Oct. (starting at wet season; *P*_spearman _= 0.041, *R*^2 ^= 0.15, linear relationship). Haplotype 112 was not observed when mean rainfall < 450 mm (*n *= 38). Here the error bar represents the frequencies for which the chance of not sampling haplotype 112 is larger than 10%. Three-year rainfall periods running from Sept. till Aug. in the case of Y-chromosomal haplotype frequencies [29]. Three-year rainfall periods running from Apr. till Mar. in the case of foetal sex ratios (in the latter case including the wet season of the focal year).

It was previously noted that the frequency of haplotype 557 per year cohort is correlated with the mean annual rainfall in the three years before birth, showing high frequencies after dry years (*P*_logisticregression _< 0.0001, years running from Sept. till Aug., *n *= 201) [29]. Additional analyses show that the same is true for haplotype 112, showing high frequencies after wet years (*P*_logisticregression _= 0.044, years running from Sept. till Aug., *n *= 201). Thus haplotypes 557 and 112 show an opposite correlation not only across seasons, but also across years (Figure 4). Again, this pattern is associated with sex-ratio variation. There was a positive correlation between the annual foetal sex ratio (years running from Nov. till Oct.) and the mean annual rainfall in the previous two or three years, including or excluding part of the wet season of the focal year (previous two years: *P*_spearman _= 0.0065-0.059, *n *= 14; for each of the four 2-year periods starting at Nov., Dec., Jan. or Feb.; previous three years: *P*_spearman _= 0.037-0.088, *n *= 14; for each of the six 3-year periods starting at Nov., Dec., Jan., Feb. Mar. or Apr.). With a rainfall period of three years including the complete wet season of the focal year (years running from Apr. till Mar.), the effect size of the sex ratio variation between dry and wet periods was 0.047 (Figure 4; *P*_spearman _= 0.041, *n *= 14), which is similar to that between seasons (rainfall > 520 mm: sex ratio = 0.531, 95% CI = 0.503-0.560, *n *= 1178; rainfall ≤ 520 mm: sex ratio = 0.484, 95% CI = 0.461-0.508, *n *= 1767). The foetal sex ratio was significantly female-biased in 1990 (*P*_Wilsonscore _= 0.0005, *n *= 332, one-sided) but significantly male-biased in 1998 (*P*_Wilsonscore _= 0.046, *n *= 114, one-sided). The average foetal sex ratio across years did not deviate from equality (*P*_exact _= 0.65, sex ratio = 0.504, 95% CI = 0.486-0.522, *n *= 2953; data pooled across years) as has also been observed in an earlier study [27].

The frequency of haplotype 557 was ≤ 0.08 (*P*_exact _= 0.90, *n *= 28, one-sided) among males conceived during the wet seasons of 1996 and 1997, but it was ≥ 0.40 (*P*_Wilsonscore _= 0.90, *n *= 38, one-sided) among those conceived between 1992 and 1994 following the severe drought of 1992 (rainfall: 243 mm vs. long-term average of 542 mm in the period 1961-2004, years running from Sept. till Aug.). This constitutes a difference of at least a factor of five in half a generation (assuming a generation time of 7.5 years [34]).

**Figure 5:**
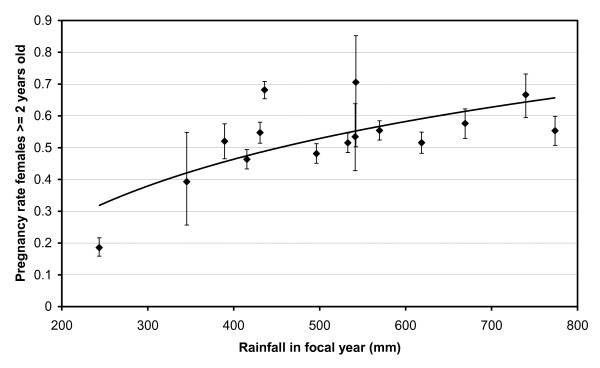
**Correlation between annual pregnancy rate and rainfall in focal year**. Error bars: 90% CI, *P*_spearman _= 0.011, *R*^2 ^= 0.51 (logarithmic relationship). Years run from Sept. till Aug.

The conception peak occurred between February and April, corresponding to 67.6% (95% CI = 65.9-69.1%, *n *= 3298) of all conceptions. It differs by one month compared to the birth peak as the gestation period is around 11 months. No more than 33.7% (95% CI = 32.1-35.3%, *n *= 3298) of the conceptions occurred during the dry season (Apr.-Oct.). There was a negative correlation between the annual pregnancy rate among ≥ 2-year old females (at the moment of sampling) and the annual rainfall (*P*_spearman _≤ 0.021, *n *= 15; for each of five 1-year periods spanning previous and focal year and starting at May, Jun., Jul, Aug. or Sept.; Figure 5 with 1-year period Sept.-Aug., *P*_spearman _= 0.011). The mean pregnancy rate was 51.6% (95% CI = 50.4-52.9%, *n *= 6439), with the yearly rate varying between 19% (1992, the driest year in the period 1961-2004) and 68% (1986, 1995, 1996). The mean adult pregnancy rate (≥ 6-year old) was as high as 69.8% (95% CI = 68.3-71.2%, *n *= 3821).

## Discussion

A plausible explanation for the associations between Y-chromosomal haplotype frequencies and foetal sex ratio is that haplotypes 112 and 557 are linked to SR genes. Haplotype 112 would be linked to a Y-chromosomal SR distorter, resulting in high population sex ratios during wet periods, and haplotype 557 to a Y-chromosomal SR suppressor, causing this haplotype to increase its frequency during dry periods when population sex ratios are low (schematic overview of the proposed mechanism in Table 2). The low sex ratios cannot be explained by mere inactivation of the SR distorter considering that they were significantly female-biased in 1990 and during the dry seasons. The SR-suppressor carrying males produce equal numbers of sons and daughters during dry periods (in dry seasons and after dry years), considering the high frequency of haplotype 557 while the sex ratio is low, but very few offspring during wet periods (in wet seasons and after wet years), as the then high sex ratio precludes a low frequency due to female-biased conception. On the other hand, SR-distorter carrying males produce substantially more sons than daughters during wet periods considering the then high frequency of haplotype 112. In turn, we argue that they produce very few offspring during dry periods considering the low frequency of haplotype 112 at those times. Although the frequency of haplotype 112 is low (0.11 after wet years and 0.18 during wet seasons), it is high enough to explain sex ratios as high as 0.53-0.55 during wet periods, assuming a frequency of 0.08-0.10 among fathers and 95% male progeny (a frequency of 0.08-0.10 among fathers results in a frequency of 0.11-0.18 among sons).

**Table 2:** Schematic overview of the effects of SR genes at conception

	Wet periods (cohorts)	Dry periods (cohorts)
**Body condition and semen quality (population mean)**	High	Low
**SR-distorter carrier (112)**	SR distorter activity ♂-biased sex ratio	Low fertility
**SR-suppressor carrier (557)**	Low fertility	SR suppressor activity 1:1 sex ratio^1^
**Wild-type (not 112 or 557)**	1:1 sex ratio	♀-biased sex ratio^2^
**Frequency haplotype 112**	↑ (SR distorter activity)	↓ (low fertility 112-carriers)
**Frequency haplotype 557**	↓ (low fertility 557-carriers)	↑ (SR suppressor activity)
**Frequency other haplotypes**	↔	↔
**Population sex ratio**	♂-biased	♀-biased

1: Suppressing a hypothesised X-chromosomal SR distorter

2: ♀-biased due to a hypothesised X-chromosomal SR distorter

↑: Increasing haplotype frequency

↓: Decreasing haplotype frequency

↔: Stable haplotype frequency

In the scenario described above, the fitness cost of temporarily reduced fertility prevents the SR distorter and SR suppressor from going to fixation. Our failure to observe haplotype 112 and 557 in dry and wet season cohorts, respectively, may even indicate a temporal reduction in fertility that nears sterility. A strong temporal reduction in fertility co-occurring with SR distortion is not implausible considering that reductions of more than 50% and even sterility have been observed with SR distorters in *Drosophila *spp., with the latter being caused by a single gene [5,11].

Female-biased sex ratios and the presence of a Y-chromosomal SR suppressor during dry periods indicate that an X-chromosomal SR distorter may also be present in KNP buffalo (Table 2). The two SR distorters and the SR suppressor may have a common origin, as it has recently been shown that a SR suppressor (*Nmy*) in *Drosophila simulans *has originated as a retro-transposed duplication of a SR distorter (*Dox*) [7,8,12]. However, we cannot exclude the possibility that the female-biased sex ratios are not due to an X-chromosomal SR distorter but instead are the result of an inherent property of Y-bearing spermatozoa making them relatively vulnerable to stress and aging [35-38]. The Y-chromosomal SR suppressor in such a case would act by making the X-bearing spermatozoa equally vulnerable to stress and aging.

The SR distorter and SR suppressor are likely to exert their effect during spermatogenesis, which is supported by two observations. Firstly, many Y-chromosomal genes are known to be involved in spermatogenesis [39-43]. They may act by influencing the transcription of various genes through epigenetic and epistatic mechanisms [7,39,44]. It is also possible that the SR distorter and SR suppressor exert their main effect post-copulation in the female genital tract by influencing sperm transport, sperm survival or fertilization capability. Secondly, in our study population it was shown that ejaculate volume, sperm motility and proportion of morphologically normal spermatozoa decrease significantly during the dry season (April-May vs. October-November) [45]. Studies on cattle (*Bos Taurus*), red deer (*Cervus elaphus*), guinea pig (*Cavia porcellus*), mice (*Mus musculus*) and humans have shown correlations between semen quality parameters on one hand and spermatozoal sex ratio, foetal sex ratio, offspring sex ratio and sex-differential fertilizing capability of spermatozoa on the other [37,38,46-50].

In the aforementioned mammals a low semen quality is associated with a low sex ratio, which also is the case for the African buffalo (i.e. low foetal sex ratio in dry season when semen quality is low). These observations may point towards a general mechanism in mammals whereby semen-quality related sex-ratio variation is driven by SR genes (distorters and suppressors). A possible explanation is that a positive association evolves more readily as males with high-quality semen may benefit from producing sons, who can inherit their father's semen quality, while males with low-quality semen benefit from producing daughters [51]. This would increase the transmission rate of newly mutated SR genes, which can be important for their invasion success as stochastic events increase the risk of establishment failure [5].

In African buffalo the association between SR genes and semen quality may have set in motion the co-evolution of Y-chromosomal and X-chromosomal SR distorters and their suppressors whose temporal variation of activity (distortion/suppression in one season, low fertility in the other) is indirectly triggered by the amount of rainfall. Here we have shown that Y-chromosomal haplotype frequencies, foetal sex ratios and pregnancy rates (indicator of female body condition) are all associated with rainfall. The decrease in semen quality in the African buffalo during the dry season is likely due to decreasing availability and quality of food resources, which are directly related to the amount of rainfall [52]. Rainfall is one of the most critical environmental factors affecting resource availability in savannah ecosystems [26,53]. In other African bovids sperm production is reduced by 30-90% during the non-breeding season [45].

The characteristics of the SR genes in African buffalo are different from those described in other species to date. The SR distorter and SR suppressor occur on a single sex chromosome and show a temporal and opposite variation in gene activity. Furthermore, there are indications that both an X-chromosomal and Y-chromosomal SR distorter are present in the population. Another unique feature is that female buffaloes in KNP are also able to influence the foetal sex in relation to body condition (in males semen quality, which likely correlates with male body condition). The effect is opposite to that of males, with females in above average body condition (although population average may be low) investing in daughters and those in below average body condition investing in sons [27].

Constant fitness models show that the co-occurrence of an X- and Y-chromosomal SR distorter can result in a stable gene polymorphism at the Y chromosome [13]. Although this only seems possible in 2% of the parameter space, extra stability may be provided by additional factors not included in the models. Such factors may be presence of a Y-chromosomal suppressor, opposite effects of the two parents on foetal sex ratio and frequency-dependent selection through an association between mating rate and sperm quality (depletion and competition). Furthermore, the constant fitness models show cycling of allele (haplotype) frequencies, which in our study population is observed in relation to rainfall.

We consider it unlikely that the postulated Y-chromosomal SR-correlated genes are associated with male mating success or semen quality (fertilization success) as such since these cannot explain sex-ratio variation. That the postulated genes exert their main effect post-conception by influencing pregnancy loss (embryonic and foetal mortality) seems equally unlikely. To prevent gene fixation, pregnancy losses with wild-type haplotypes should be similar to those with haplotypes 112 and 557, which would have to be considerable (at least 50%) to explain the large haplotype frequency variations (up to a factor of five). Moreover, additional losses of female embryos and foetuses must be assumed to explain the male-biased sex ratios. However, high pregnancy losses are difficult to reconcile with adult pregnancy rates of 70%. Furthermore, this scenario would imply that haplotype 557 is associated with below average pregnancy loss during dry periods while the opposite is true for the remaining haplotypes, as indicated by the then low pregnancy rates and conception numbers for the population as a whole. It is difficult to envisage a physiological mechanism capable of making haplotype 557 so successful under stressful conditions (dry periods), yet so unsuccessful under beneficial conditions (wet periods). Cryptic female choice at least seems unlikely since this would imply a positive adaptive value of choosing male offspring on the basis of just a single Y-chromosomal haplotype, unlinked to the autosomal genome. Furthermore, haplotype-specific pregnancy loss is difficult to relate to frequency-dependent selection. This would require that the physiological mechanism by which the haplotypes are associated with pregnancy loss in individual females is modulated, directly or indirectly, by their frequency in the population at large. A final alternative explanation would be a co-occurrence of two independent mechanisms: rainfall-dependent and haplotype-specific mating or fertilization success in the fathers on one hand and rainfall-dependent and sex-specific pregnancy loss in the mothers on the other. Again, this scenario is difficult to relate to frequency-dependent selection while it also requires a mechanism that causes haplotype 557 to be most successful under stressful conditions but least successful under beneficial conditions.

Although we consider the alternative explanations for the correlations between Y-chromosomal haplotype frequencies and foetal sex ratio unlikely, and propose the presence of SR genes as the most plausible explanation, this will need direct confirmation in a future studies. This may be done by analyzing associations between semen quality and spermatozoal sex ratio in individual bulls with known Y-chromosomal genotype.

## Conclusions

Polymorphic active SR genes in natural populations of mammals have rarely been observed but may be more common than previously thought. We suggest that in many mammals semen-quality related primary sex-ratio variation is influenced by SR genes. Stable gene polymorphism may be maintained with relative ease when SR genes fulfil one or more of the specific characteristics observed in African buffalo.

## Methods

### Sample data

From 1969 onwards, culling of African buffalo has been used in KNP as a population management and disease monitoring tool [27,54]. The data are from 3184 foetuses and their mothers collected during culling events in 1978 and between 1984 and 1996 (data from SANParks Scientific Services Department), mostly between May and November (99.4%), from 218 0-1 year-old calves and from 114 foetuses and their mothers culled from 32 herds between September and November 1998, and from published data from 43 0-4 month-old calves culled between June 1982 and February 1983 [27,33,54].

The foetuses from 1998 are the same as those reported previously [27]. Foetus sex was determined using external genitalia characteristics. Sexed foetuses ≤ 18 g (age ± 70 days, *n *= 150) were excluded from the data analyses because they appeared to be outliers, considering that they were predominantly diagnosed as male, irrespective of season or year (76% males, 114/150; 95% CI: 69-82%). This is most likely to be attributed to sexing errors and ascertainment bias. In the closely related Asian buffalo (*Bubalus bubalis*) reliable foetal sex differentiation is possible no sooner than the 56^th ^day of pregnancy with macroscopic diagnosis and the 70^th ^day of pregnancy with ultrasonographic diagnosis, with external genitalia occurring sooner in males than in females [55,56]. Furthermore, 195 foetuses from 1978-1996 > 18 g were of unknown sex, leaving 2839 foetuses from 1978-1996 and 114 foetuses from 1998 for sex-ratio analyses. The sex ratio of these foetuses did not significantly deviate from equality (see Results section) indicating that there was no systematic bias in foetus sexing. Conception dates were assigned using a weight-at-age regression (8.57*weight (g)^1/3 ^+ 48.5) [57].

The age of the animals was estimated in years on the basis of dental wear patterns, number of erupted incisor teeth, body size, and horn development [27]. The age of the calves from 1998 was estimated in 3-month and half-year cohorts (age categories: 0-3 months, *n *= 5; 3-6 months, *n *= 63; 6-9 months, *n *= 43; 9-12 months, *n *= 5; 1-1.5 year, *n *= 73; 1.5-2 year, *n *= 29). Conception dates of the calves were estimated on the basis of the midpoint age per age cohort (i.e. 1.5, 4.5, 7.5, 10.5, 15 and 21 months for the 1998 data and 2 months for the 1982-1983 data), assuming a gestation period of 340-343 days [25,58].

### Genetic analyses

Y-chromosomal microsatellite marker data were available from the males culled in 1998, consisting of 74 0-1 year-old calves and 127 subadults and adults (≥ 2 years old). In an earlier study, which gives detailed information on the molecular analyses, these data were used to assess the genetic diversity per year cohort [29]. Three microsatellites were analyzed whose combination of alleles constitute a haplotype, since the Y chromosome is a single, haploid non-recombining unit (except for a small pseudoautosomal region). A 3-number code was assigned to each haplotype, with each number corresponding to a microsatellite allele. Fifteen haplotypes were observed in total. No foetus samples were available for DNA analyses since they were not stored after analysis in the field. Here we used the microsatellite data to test for genetic differentiation among season cohorts in calves (Figure 1). Randomisation-based tests for genetic differentiation with 100,000 repetitions and no pooling of haplotypes were performed using FSTAT 2.9.3.2 [59,60]. The program generates contingency tables of haplotypes by samples (age cohorts) and the statistic that is used to classify them is the log-likelihood statistic G.

### Statistical analyses

Effect sizes were estimated as the absolute difference between sex ratios, which is defined as the proportion of males. Confidence intervals of the sex ratios were estimated using the Wilson score formula [61]. The significance level of differences between two sex ratios or haplotype frequencies was estimated with the Fisher's exact test. Haplotype frequencies were regressed against rainfall in forward and backward stepwise (conditional) logistic regression models using SPSS 15.0.1.1. Correlations were analysed using the Spearman rank test. All significance tests in this study were performed two-sided, unless otherwise indicated.

### Precipitation

Monthly rainfall data for September 1960 to December 2004 were averaged across 13 South African Weather Service (SAWS) rainfall stations in KNP. The body condition of adult female buffalo in KNP coincides with monthly Normalized Difference Vegetation Index (NDVI) values, which lags the rainfall by one month and which is a proxy for vegetation productivity [26]. NDVI values tend to increase sharply in November and reach their peak in February-March [26]. The wet season was therefore defined as the period from November to March. During this period 78% of the rainfall occurs, which is five times higher on a monthly basis than in the dry season.

The raw data used in this study are available in an additional file (Additional file 1).

## Authors' contributions

PvH designed and performed the molecular DNA analyses, analyzed the data and wrote the paper. PDvH and ADSB contributed samples. ADSB contributed laboratory reagents and facilities. All authors discussed and commented on the paper.

## Supplementary Material

Additional file 1 **Raw data**. Additional file 1 (Microsoft Office Excel^®^) contains raw data from the animals culled between 1978 and 1998, data on Y-chromosomal genetic diversity from the animals culled in 1998 and SAWS rainfall data. Click here for file
